# A novel "pearl box" cataract associated with a mutation in the connexin 46 (*GJA3*) gene

**Published:** 2007-06-04

**Authors:** Kamlesh Guleria, Vanita Vanita, Daljit Singh, Jai Rup Singh

**Affiliations:** 1 Centre for Genetic Disorders, Guru Nanak Dev University, Amritsar, India; 2 Dr. Daljit Singh Eye Hospital, Amritsar, India

## Abstract

**Purpose:**

To undertake mutation screening in the connexin 46 (*GJA3*) gene in seven congenital cataract families of Indian origin.

**Methods:**

Seven Indian families with congenital cataract were analyzed by detailed family history and clinical evaluation. Each family had two to five affected members. Mutation screening was carried out in the candidate gene, connexin 46 (*GJA3*), using bidirectional sequencing of amplified products. Segregation of the observed change with the disease phenotype was further tested by restriction fragment length polymorphism (RFLP).

**Results:**

Sequencing of the coding region of *GJA3* showed the presence of a novel, heterozygous C260T change in one family (CC-472) who had two affected members. The cataract phenotype gave the appearance like a "pearl box" in these two affected individuals of this family. The observed C260T substitution created a novel restriction enzyme site for *Nla*III and resulted in substitution of highly conserved threonine at position 87 by methionine (T87M). *Nla*III restriction digestion analysis revealed this nucleotide change was not in unaffected members of this family or in 100 unrelated control subjects (200 chromosomes) with the same ethnic background.

**Conclusions:**

This is a novel mutation identified in the second transmembrane domain of the connexin 46. These findings thus expand the mutation spectrum of the *GJA3* in association with congenital cataract.

## Introduction

Congenital cataract is one cause of childhood blindness worldwide. It is clinically and genetically a highly heterogeneous lens disorder, with autosomal dominant inheritance being most common. Congenital cataract can occur either as an isolated anomaly or in association with other ocular anomalies or as a component of multisystemic disorders. Its incidence is estimated to be between 2.2 and 2.49 per 10,000 infants [1,2]. In one-third of the cases worldwide, congenital cataract has been reported as familial [3,4]. At least 35 loci and mutations in 15 genes have been identified as being involved in the pathogenesis of various forms of congenital and developmental cataracts [5].

The eye lens, an avascular organ, is highly dependent on intercellular communication for volume regulation and metabolic homeostasis [6]. This is achieved by cell-to-cell communication via gap junctions, which are encoded by the connexin genes. These gap junctions facilitate the exchange of ions, metabolites, signaling molecules, and other molecules that have a molecular weight up to 1 kDa between adjacent cells [7]. In humans, more than 20 genes coding connexins of varying molecular mass ranging between 25-62 kDa have been identified. Three of these, connexin 43, connexin 46, and connexin 50, are expressed in the lens [8]. Mutations in either connexin 46 or in connexin 50 have so far been linked with congenital cataract [9,10].

The aim of present study was to identify the mutations in the connexin 46 (*GJA3*) gene in seven congenital cataract families of Indian origin. Upon sequence analysis of the *GJA3*, we identified a heterozygous C260T change resulting in the substitution of a highly conserved threonine by methionine at codon 87 (T87M) in the affected individuals of one family (CC-472). The change cosegregated completely with the disease phenotype. This is a novel mutation and has not been reported previously with congenital cataract.

## Methods

### Clinical evaluation and collection of genetic material

The present study was undertaken in collaboration with Dr. Daljit Singh Eye Hospital, Amritsar, Punjab (India). The study protocols adhered to the tenets of the Declaration of Helsinki and were approved by the institutional review board of Guru Nanak Dev University, Amritsar. A pediatric ophthalmologist performed a detailed clinical examination on each proband and selected relatives. The exam included Snellen visual acuity, A-scan ultrasonography, and slit-lamp examination with pupil dilation. The phenotypes were documented using slit-lamp photography. A detailed family history and pedigree were recorded for each case. In the present study we analyzed seven congenital cataract families, each of whom had two to five affected members. Four families had the autosomal dominant mode of inheritance and three presented with the autosomal recessive inheritance pattern. After affected and unaffected family members gave informed consent, 5-10 ml venous blood was drawn, and DNA was extracted for subsequent molecular genetic analysis.

### PCR and DNA sequencing

*GJA3* (GenBank NM_021954), located at 13q11 and consisting of a single coding exon encoding 435 amino acids, was sequenced using previously published primer sequences [11]. Genomic DNA from two affected and one unaffected individual from each family were amplified. PCR and sequencing reactions were performed following conditions detailed elsewhere [12,13]. Electrophoresis of purified sequencing reaction products was performed on 5% urea-polyacrylamide gel on ABI Prism 377 DNA sequencer (Applied Biosystems, Foster City, CA), and data was analyzed using sequence analysis software version 3.4.1 (Applied Biosystems).

### Restriction endonuclease analysis

The DNA fragment harboring the mutation was amplified for both affected and unaffected family members, and PCR products were digested with *Nla*III restriction enzyme following directions given by the manufacturer (New England Biolabs, Beverly, MA). Restriction digestion products were analyzed on a 2.5% agarose gel. In addition, 100 unrelated controls from similar ethnic background were also similarly tested.

## Results

**Figure 1:**
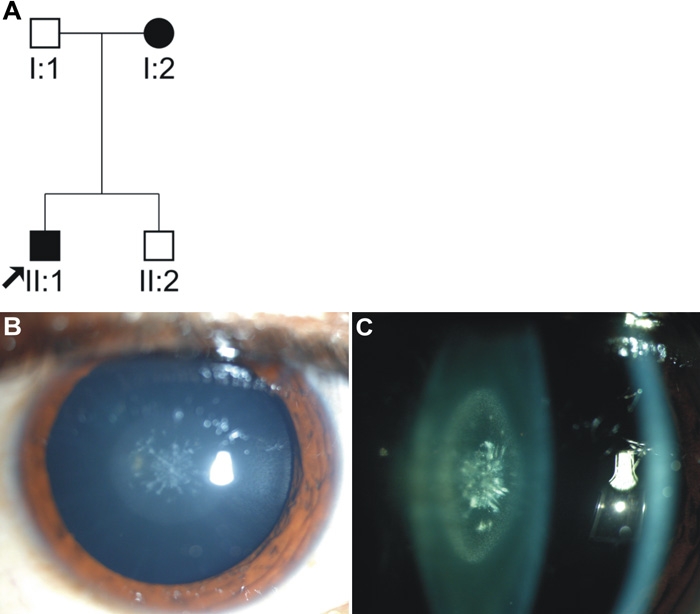
Pedigree of family CC-472 and lens photograph of proband. **A**: Pedigree of family CC-472 with two affected individuals: mother (I:2) and son (II:1). Circle represents females while squares indicate males. Shaded shapes indicate affected individuals. **B**: Oblique illumination of phenotype (II:1) showing white spots of different sizes and shapes in the fetal nucleus. **C**: Optical section showing wide surface opacity of the fetal nucleus composed of fine white spots. The space between the surface opacity and central white spots is optically empty.

In the present study, four families with autosomal-dominant congenital cataract and three families with autosomal-recessive congenital cataract were investigated. Each family had 2-5 affected members with different clinical morphologies (one family each with pearl box, posterior cortical, granular, absorbed membranous, and round ball cataract and two autosomal-recessive families with posterior subcapsular type cataract). Mutation analyses of the connexin 46 (*GJA3*) gene in these seven families revealed a novel heterozygous mutation in only one family (CC-472). In this family, a son and his mother were affected with bilateral congenital cataract (Figure 1A). The opacities involved both the embryonal and fetal nuclei separated by a clear space. Fine white spots made up the opacity of the fetal nucleus (Figure 1B). The embryonal nucleus was composed of coarse white spots of various sizes that were round when located toward the center and linear-shaped in the periphery. There was an optically empty space between the embryonal nuclear and fetal nuclear opacities. The peripheral zone outside the fetal nucleus was clear. The cataract phenotype gave the appearance of a box produced by the fetal nuclear opacities and pearls by the embryonal nuclear opacities (Figure 1C). Hence, this phenotype was termed "pearl box" cataract.

**Figure 2:**
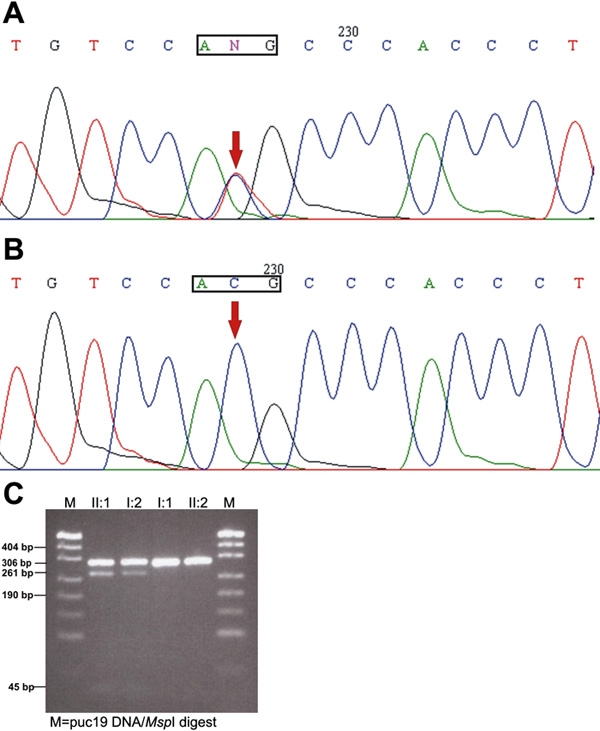
Mutation analysis of family CC-472. **A**: DNA sequence of a portion of *GJA3*, showing the heterozygous 260C>T transition that changes threonine to methionine at codon 87 in affected individual (II:1). **B**: DNA sequence electropherogram of unaffected individual (I:1). **C**: *Nla*III restriction digestion analysis of amplified DNA at the mutation site. The 308 bp PCR product is cleaved into four fragments (306 bp, 261 bp, 45 bp, and 2 bp) in affected individuals (I:2 and II:1) and into two fragments (306 bp and 2 bp) in unaffected individuals (I:1 and II:2).

Direct sequencing of *GJA3* revelaed a novel heterozygous C>T transition (Figure 2A) at position 260 (c. 260C>T) in the affected individuals of CC-472 family. It is this transition that led to the replacement of highly conserved threonine with methionine at codon 87 (Thr87Met). This substitution created a novel *Nla*III restriction site that segregated completely with the disease phenotype in this family (Figure 2C). This nucleotide change was not observed in the 100 control subjects from similar ethnic background tested by restriction digestion analysis (data not shown). Testing of six additional families revealed no disease-linked change or any polymorphic change in *GJA3*.

## Discussion

**Figure 3:**
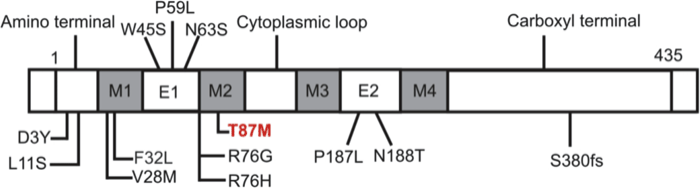
Schematic diagram of the connexin 46 polypeptide and locations of identified mutations. Connexin 46 polypeptide (435 amino acids) has nine structural domains including a cytoplasmic NH_2_-terminus, four transmembrane domains (M1-M4), two extracellular loops (E1-E2), a cytoplasmic loop, and a cytoplasmic COOH-terminus (modified figure from Bennett et al. [31]). The relative location of the T87M mutation (indicated in red) and other reported mutations associated with congenital cataracts in humans are marked.

We report a novel change, T87M in the connexin 46 gene, in one family (CC-472) who has what we term "pearl box" cataract. The T87M substitution is likely to be disease causative as it segregated among the affected members only and was not detected either in unaffected family members or in 100 unrelated controls. The 260C>T substitution observed in the present study resulted in the replacement of polar amino acid threonine (T) with nonpolar amino acid methionine (M) at codon 87 (T87M) localized in the second transmembrane domain (M2) of the connexin 46 polypeptide (Figure 3). This is the first identified mutation that lies within in the second transmembrane domain (M2) of the connexin 46 polypeptide associated with novel "pearl box" cataract.

**Figure 4:**
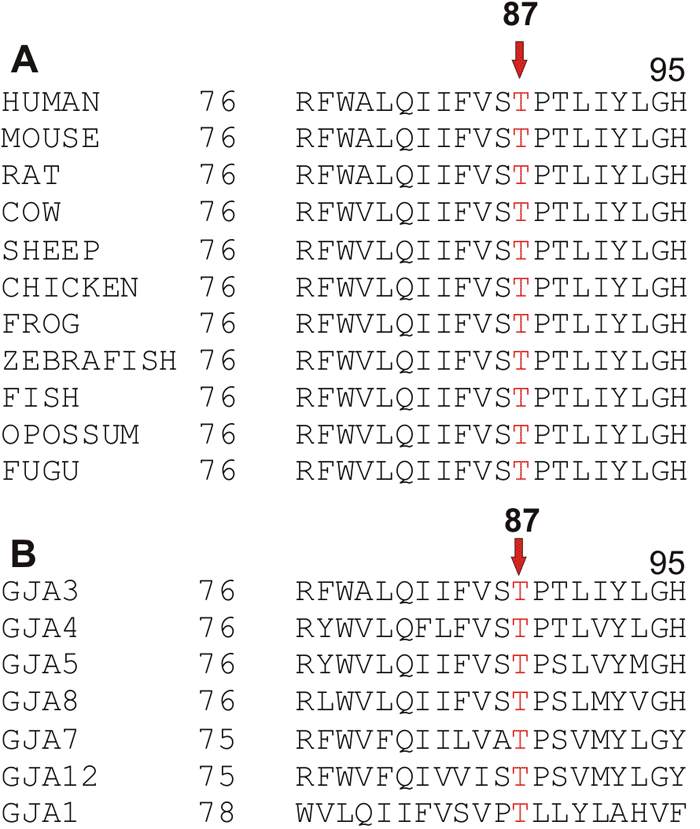
A multiple sequence alignment of amino acid sequences of connexin 46 in different species and in different human alpha-connexins. Alignment data indicate that threonine is highly conserved in different species (**A**) and in different human alpha-connexins (**B**). Arrow points to the conserved threonine at position 87, which is marked in red.

A multiple sequence alignment of the amino acid sequences of connexin 46 showed that threonine at position 87 is phylogenetically conserved in the second transmembrane domain of connexin 46 in different species (Figure 4A) and also in different human α-connexins (Figure 4B). This indicates that the threonine at position 87 in connexin 46 is likely to be functionally important and the T87M mutation may therefore has a detrimental physiological effect. In the connexin 50 (*GJA8*) gene localized at 1q21, corresponding mutations (P88S and P88Q) in the second transmembrane domain (M2) resulting in zonular pulverulent [14] and lamellar pulverulent cataract [15], respectively, have been reported. Pal et al. [16] further reported that P88S mutant connexin 50 abolishes the function of the gap junction channel and acts in a dominant negative manner. In humans, P87S and P87A mutations located within M2 of the connexin 32 (*GJB1*) gene have been reported in association with Schwann cell dysfunction and peripheral nerve degeneration in X-linked Charcot-Marie-Tooth disease [17-19]. The authors hypothesized that these mutations (P87S and P87A) in M2 of the connexin 32 polypeptide may impair voltage-dependent opening and closing of gap junctions in electrically excitable tissues. Suchyna et al. [20] have also demonstrated that in the connexin 26 polypeptide proline at codon 87 has a function in voltage-gating of gap junctions. Although, the effect of T87M mutation observed in the present study on the activity of the connexin 46 has not been directly tested, we speculate that, like P88S in the *GJA8* and other dominantly inherited mutations reported in different connexins, this mutation also results in inappropriate association of connexins and alters the function of endogenous wild-type connexins in the affected individuals in a dominant negative way.

Defects in the connexin 46 and connexin 50 genes have been reported to cause cataract in mice. Point mutations A47C and V64A in the connexin 50 gene have been reported to result, respectively, in nuclear opacities (*No2*) and nuclear with posterior suture opacities (*Aey5*) in mice [21,22]. Gong et al. [23] reported that mice homozygous for disrupted connexin 46 developed nuclear cataracts due to failure in maintenance of differentiation and of crystallins solubility, while connexin 50 knockout mice had reduced ocular growth along with nuclear cataract [24,25].Targeted replacement of connexin 50 with connexin 46 in mice has revealed the role of connexin 50 in lens and eye growth and that of connexin 46 in maintaining differentiation by nonspecific restoration of intercellular communication [26].

The cataract phenotypes that are reported to be linked with the *GJA3* mutations share genotype-phenotype similarities to some extent, but they also exhibit some differences with respect to the appearance and location of opacities within the lens. At this point, 12 mutations in *GJA3* have been reported to be associated with autosomal-dominant congenital cataract in humans involving different domains of connexin 46 polypeptide (Table 1). Most of the cataract phenotypes linked with mutations in the *GJA3* are of nuclear or zonular pulverulent types. The phenotype observed in present study (CC-472 family) is different in its appearance from the earlier reported types (Table 1) as it appears like pearls in a box (Figure 1B,C). The differences in the morphologies of cataract phenotypes associated with mutations in the *GJA3* in different families may be attributed to the action of modifier genes or environmental factors that could affect the expression of the connexin 46 gene and hence resulting cataract types.

**Table 1:** Reported mutations in *GJA3* associated with different congenital cataract phenotypes in different families.

**Amino acid change**	**Location**	**Cataract type**	**Phenotype description**	**Family origin**	**Reference**
D3Y	-NH2 terminal cytoplasmic loop	Zonular pulverulent	Progressive zonular pulverulent cataract	Flispanic	[27]
L11S	-NH2 terminal cytoplasmic loop	Ant-egg	Lamellar cataract with dense ant-egg like structures imbedded in the lens, primarily confined to the perinuclear layers and to lesser degree in the fetal nucleus	Danish	[9]
V28M	First transmembrane domain	Variable	Variable cataract types like total, anterior capsular cataract with posterior cortical opacities in different individuals	Indian	[28]
F32L	First transmembrane domain	Nuclear pulverulent	Punctate opacities in the central zone of the lens limited to the embryonal nucleus	Chinese	[29]
W45S	First extracellular loop	Nuclear	Progressive nuclear cataract	Chinese	[30]
P59L	First extracellular loop	Nuclear punctate	Coarse punctate opacities located in the central or nuclear region of the lens	American	[31]
N63S	First extracellular loop	Zonular pulverulent	Coarse and granular opacities in the central zone of the lens. Fine dust-like opacities predominated in die peripheral zone of the lens.	Caucasian	[11]
R76G	Boundary of first extracellular loop and second transmembrane domain	Total	Total leas opacification	Indian	[28]
R76H	Boundary of first extracellular loop and second transmembrane domain	Nuclear pulverulent	Faint lamellar nuclear opacity surrounding pulverulent nuclear opacities, some with fine gold dots or haze and some with needle-like peripheral riders	Australian	[32]
T87M	Second transmembrane domain	Pearl box	A bunch of white spots seen in the embryonal nucleus. The central white spots distributed in a radial manner. The space between the surface opacity and central white spots is optically empty. Surface opacity gives the appearance of a box while central white spots as of pearls in it.	Indian	Present study
P187L	Second extracellular loop	Zonular pulverulent	Central dust-like opacity affecting the embryonal, fetal and infantile nucleus of the lens surrounded by snowflake-like opacities in the anterior and posterior cortical region of the lens	Caucasian	[33]
N188T	Second extracellular loop	Nuclear pulverulent	Progressive, central pulverulent opacity affecting the embryonal, fetal and infantile nucleus of the lens	Chinese	[34]
S380fs	-COOH terminal cytoplasmic loop	Zonular pulverulent	Coarse and granular opacities in the central zone of the lens. Fine dust-like opacities predominated in die peripheral zone of the lens.	Caucasian	[11]

Mutation spectrum of the connexin 46 (GJA3) gene and cataract phenotypes in different congenital cataract families.

In summary, we describe a novel heterozygous T87M mutation in the connexin 46 polypeptide associated with "pearl box" cataract. On the basis of observed phenotypic as well as genotypic variability as compared to previously published reports, the present study further expands the genetic and phenotypic heterogeneity of congenital cataract.
